# PKC regulates αKlotho gene expression in MDCK and NRK-52E cells

**DOI:** 10.1007/s00424-023-02863-3

**Published:** 2023-09-29

**Authors:** Lisa Wolf, Julia Vogt, Jana Alber, Domenic Franjic, Martina Feger, Michael Föller

**Affiliations:** 1 Department of Physiology, University of Hohenheim, Garbenstraße 30, 70599 Stuttgart, Germany; 2 Core Facility Hohenheim, Data and Statistical Consulting, University of Hohenheim, 70599 Stuttgart, Germany

**Keywords:** Phorbol ester, Phosphate, Longevity, FGF23

## Abstract

Particularly expressed in the kidney, αKlotho is a transmembrane protein that acts together with bone hormone fibroblast growth factor 23 (FGF23) to regulate renal phosphate and vitamin D homeostasis. Soluble Klotho (sKL) is released from the transmembrane form and controls various cellular functions as a paracrine and endocrine factor. αKlotho deficiency accelerates aging, whereas its overexpression favors longevity. Higher αKlotho abundance confers a better prognosis in cardiovascular and renal disease owing to anti-inflammatory, antifibrotic, or antioxidant effects and tumor suppression. Serine/threonine protein kinase C (PKC) is ubiquitously expressed, affects several cellular responses, and is also implicated in heart or kidney disease as well as cancer. We explored whether PKC is a regulator of αKlotho. Experiments were performed in renal MDCK or NRK-52E cells and PKC isoform and αKlotho expression determined by qRT-PCR and Western Blotting. In both cell lines, PKC activation with phorbol ester phorbol-12-myristate-13-acetate (PMA) downregulated, while PKC inhibitor staurosporine enhanced αKlotho mRNA abundance. Further experiments with PKC inhibitor Gö6976 and RNA interference suggested that PKCγ is the major isoform for the regulation of αKlotho gene expression in the two cell lines. In conclusion, PKC is a negative regulator of αKlotho gene expression, an effect which may be relevant for the unfavorable effect of PKC on heart or kidney disease and tumorigenesis.

**Supplementary Information:**

The online version contains supplementary material available at 10.1007/s00424-023-02863-3.

## Introduction

αKlotho is mainly expressed in the kidney as a transmembrane protein [33, 36]. On the one hand, it enhances affinity of fibroblast growth factor 23 (FGF23) for fibroblast growth factor receptor (FGFR) proteins, thereby functioning as a co-receptor [59]. FGF23 is a hormone predominantly produced by bone cells under physiological conditions that is a major regulator of vitamin D and phosphate homeostasis [53] along with other hormonal regulators of Ca^2+^ and phosphate metabolism, especially 1,25(OH)_2_D_3_ (biologically active vitamin D) and parathyroid hormone [3, 5, 20]. The joint action of FGF23 and its co-receptor αKlotho results in a decrease of membrane abundance of NaPi-IIa, the major Na^+^-dependent phosphate transporter of the renal proximal tubule [23], and in lower expression of 1α-hydroxylase, the key enzyme for the synthesis of 1,25(OH)_2_D_3_ in the kidney and in other cells [1, 51]. Hence, FGF23 along with αKlotho lowers the blood levels of both, phosphate and 1,25(OH)_2_D_3_ [54].

Apart from transmembrane αKlotho, a circulating form of this protein exists named soluble Klotho (sKL). SKL has paracrine and endocrine effects on various cells including the regulation of ion transport or significant signaling pathways such as Wnt or phosphoinositide-3-kinase (PI3K) signaling [29, 63, 75].

Lack of either αKlotho or FGF23 leads to a strong phenotype in mice that reminds of aging of human beings: The animals exhibit aging-related disorders of most organs and tissues and die prematurely [37, 55]. Above all, organs and tissues are affected by strong calcification [69] that can be prevented if the animals are fed a low vitamin D- or low phosphate diet [8, 46]. Hence, premature aging in αKlotho or FGF23 deficiency is dependent on deranged phosphate metabolism [32, 49].

Conversely, mice have a strongly expanded life span if αKlotho is overexpressed, a finding corroborating the role of αKlotho as an antiaging factor [37].

In addition, αKlotho has been demonstrated to have beneficial effects on multiple organs and to favorably influence a broad range of diseases: It is cardio- or nephroprotective [4, 19, 72], it provides protection from neurodegeneration, it exerts anti-inflammatory, antifibrotic, and antioxidant effects, and it has turned out to be a tumor suppressor [43, 44, 73, 74, 77]. Hence, lower αKlotho levels result in a poorer prognosis of cardiovascular or renal diseases as well as different human malignancies [14, 42, 68]. Also, FGF23 is a marker of kidney function [9, 26] or cardiovascular disease [12] with higher FGF23 levels predicting worse outcome.

Protein kinase C (PKC) is expressed in every cell and among the major regulators of most cellular functions and responses [10]. It is a serine/threonine kinase and classically activated by Ca^2+^ and diacylglycerol (DAG) that is released from the membrane in response to phospholipase C activation by G_q_ protein-coupled receptors or receptor tyrosine kinases (classical PKC) [31, 58]. Novel PKC isoforms are activated by DAG only whereas atypical PKC isoforms depend on other mechanisms for activation [28].

On a cellular level, PKC is a regulator of proliferation, migration, and apoptosis [16, 52]. Similar to αKlotho, PKC isoforms are implicated in neurodegeneration and different human diseases [27]. PKC is a regulator of FGF23 [2, 22]. Whereas αKlotho is a tumor suppressor, PKC rather promotes tumorigenesis [25]. PKC is also relevant for kidney disease including diabetic nephropathy [64] and various heart diseases [57].

Since αKlotho and PKC are relevant for similar pathologies and are often characterized by opposing effects, we wondered whether PKC is a regulator of αKlotho expression. We therefore performed experiments in Madin-Darby canine kidney (MDCK) and normal rat kidney (NRK-52E) cells to elucidate the effect of PKC on αKlotho and to identify the PKC isoform involved.

## Methods

### Cell culture

MDCK (NBL-2) cells (CVCL_0422; Cell Lines Services, Eppelheim, Germany) were cultured in Dulbecco’s modified Eagle medium F12 (DMEM/F-12; Gibco, Life Technologies, Darmstadt, Germany) with additional 5% fetal bovine serum (Gibco), 100 U/ml penicillin, 100 μg/ml streptomycin (Gibco), and 2 mM glutamine (Gibco). NRK-52E cells (ECACC 87012902; European Collection of Authenticated Cell Cultures, Salisbury, UK) were cultured in Dulbecco’s modified Eagle medium (DMEM) plus 5% newborn calf serum, 100 U/ml penicillin, and 100 μg/ml streptomycin (Gibco). For experiments, cells were plated at a density of 1.5×10^5^ cells per well (MDCK) or 2×10^5^ cells per well (NRK-52E) in 6-well plates at 5% CO_2_ and 37°C. After 24 h, cells were treated with PKC activator phorbol-12-myristate-13-acetate (PMA) (0.01–600 nM as indicated; Peprotech, Hamburg, Germany) and/or PKC inhibitors staurosporine (0.01–1 nM as indicated; Cayman Chemicals, Ann Arbor, MI, USA), 300 nM sotrastaurine (United States Biological, Salem, MA, USA), or 40 nM Gö6976 (Biomol, Hamburg, Germany) for further 24 h. Control cells were incubated with the respective amount of solvent dimethyl sulfoxide (DMSO; AppliChem, Darmstadt, Germany) for all experiments.

### Silencing

MDCK cells (1.2×10^5^ per well) were cultured for 24 h in complete growth medium. Cells were then transfected with custom-designed PKCγ small interfering RNA (siRNA) (25 nM; sense: ACAAGUUACUGAACCAGGAtt, antisense: UCCUGGUUCAGUAACUUGUAC, Invitrogen, Thermo Fisher Scientific, Darmstadt, Germany), custom-designed PKCη siRNA (25 nM; sense: GAAAUGGGAUCGGAGUUAAtt, antisense: UUAACUCCGAUCCCAUUUCcc, Invitrogen) or 25 nM non-targeting control siRNA (#4390843, Invitrogen) using 5-μl Lipofectamine RNAiMAX (Invitrogen) transfection reagent in antibiotic-free complete medium for 24 h. For silencing of NRK-52E cells, 1.5×10^5^ cells per well were grown in complete medium for 24 h and then transfected with custom-designed PKCγ siRNA (50 nM; sense: ACAAGUUACUGAACCAGGAtt, antisense: UCCUGGUUCAGUAACUUGUAC, Invitrogen) or 50 nM non-targeting control siRNA (#4390843, Invitrogen) using 5 μl DharmaFECT 1 (Horizon Discovery, Cambridge, UK) transfection reagent in antibiotic-free complete medium for 24 h. Silencing efficiency was determined by quantitative real-time PCR.

### Quantitative real-time PCR (qRT-PCR)

Cells were washed with ice-cold PBS and total RNA was isolated using TriFast (Peqlab, Erlangen, Germany). Isolates from MDCK cells were subjected to DNase digestion and RNA was extracted using NucleoSpin RNA Mini kit (Macherey-Nagel, Düren, Germany). Total RNA (1.2 μg; 60 ng/μl) was taken for cDNA synthesis with random primers and the GoScript Reverse Transcription System (both from Promega, Mannheim, Germany). Quantitative RT-PCR was performed on a CFX Connect Real Time System (Bio-Rad Laboratories, Feldkirchen, Germany) utilizing GoTaq qPCR Master Mix (Promega). Conditions were: 95°C for 5 min; followed by 40 cycles of 95°C for 10 s, annealing at primer-specific temperature for 30 s, and 72°C for 30 s. The final volume of the qRT-PCR master mix was 20 μl comprising 2 μl cDNA, 0.5 μM (or 0.25 μM for rat and dog αKlotho) forward and reverse primer, and water. Primer sequences are listed in Table 1.

**Table 1:** List of primers used for qRT-PCR

Species	Gene	Forward primer (5′→3′)	Reverse primer (5′→3′)	Reference sequence	Annealing temp.
*dog*	*TBP*	CCTATTACCCCTGCCACACC	GCTCCCGTACACACCATCTT	XM_038684469.1	60°C
*dog*	*KL*	AAATGAAGCTCTGAAAGCC	AATGATAGAGGCCAAACTTC	XM_038434663.1	56°C
*dog*	*PRKCG*	ATCATGGAACAAACAGTCAC	CTTGGTTAGGAACCCCTTG	XM_038655881.1	56°C
*dog*	*PRKCA*	AGCTCCACGTCACTGTACGA	GGGGATTCAGTGTAGAGCGG	XM_038676073.1	56°C
*dog*	*PRKCB*	GAACGAAACATTCAGATTCC	TCCCAATCCCAAATCTCTAC	XM_038668826.1	56°C
*dog*	*PRKCD*	GGCATTGAACCAAGTGACCC	TACGTCCCGTTGTCTTCACCT	XM_038426494.1	56°C
*dog*	*PRKCE*	GACTTTCCTCCTCGACCCCT	GCTCCAGATCAATCCAGTCC	XM_846768.6	56°C
*dog*	*PRKCH*	CAGGGAGTTTATCTGGGGAGT	ACACTGAAGTCCTTGTCGCA	XM_038673803.1	56°C
*dog*	*PRKCI*	CAGTCACCCATGCCTTCAGA	CCCTGGTATTCATTGCCTCCT	XM_022414139.2	62°C
*dog*	*PRKCQ*	TGCTAATGAATGCCAGATAC	AAAAGCAAAGAAGCCTTCAG	XM_038658475.1	56°C
*dog*	*PRKCZ*	GGTGGACAATGAAGGTGACCC	ACACGTGAATGATGAGCCCT	XM_005620419.4	62°C
*rat*	*Tbp*	ACTCCTGCCACACCAGCC	GGTCAAGTTTACAGCCAAGATTCA	NM_001004198.1	57°C
*rat*	*Kl*	CAACTACATTCAAGTGGACC	CAGTAAGGTTTTCTCTTCTTGG	NM_031336.2	54°C
*rat*	*Prkcg*	TGTGGAACGAGACCTTCGTG	AAACTTCTGGAGGAGGCTGC	XM_047439092.1	59°C
*rat*	*Prkca*	CTGAACCCTCAGTGGAATGAGT	GGCTGCTTCCTGTCTTCTGAA	NM_001399299.1	61°C
*rat*	*Prkcb*	AACGGCTTGTCAGATCCCTA	CCACTCCGGCTTTCTGTAGT	XM_039101236.1	61°C
*rat*	*Prkcd*	TGTGAAGACTGCGGCATGAA	AGGTGAAGTTCTCAAGGCGG	NM_133307.2	60°C
*rat*	*Prkce*	CGAGGACGACTTGTTTGAATCC	CAGTTTCTCAGGGCATCAGGTC	NM_017171.2	61°C
*rat*	*Prkch*	CAGGGAGTTTATCTGGGGAGT	ACACTGAAGTCCTTGTCGCA	XM_038673803.1	56°C
*rat*	*Prkci*	CTCCTGATCCACGTGTTCCC	GGATGACTGGTCCATTGGCA	NM_032059.1	61°C
*rat*	*Prkcq*	TGCCGACAATGTAATGCAGC	ACACTTGAGACCTTGCCTCG	NM_001276721.1	61°C
*rat*	*Prkcz*	GTGGACCCCACGACAACTT	GATGCTTGGGAAAACGTGGA	NM_022507.1	60°C

Calculated mRNA expression levels were normalized to TATA box-binding protein (TBP) transcript levels. TBP mRNA expression was not significantly affected by either treatment. Relative quantification of target gene expression was accomplished by the 2^−ΔCT^ method.

For qualitative mRNA expression analysis of PKC isoforms in MDCK cells, a minus reverse transcriptase (−RT) control was included in all qRT-PCR assays. About 10 μl of the qRT-PCR product as well as non-template control and −RT control were loaded onto a 1.5% ethidium bromide agarose gel. Bands were visualized by UV spectrometry (BioDocAnalyzer, Biometra, Göttingen, Germany).

### Cell viability assay

Cell viability was determined by 3-[4,5-dimethylthiazol-2-yl]-2,5-diphenyltetrazolium bromide (MTT) assay. Cells were cultured for 24 h and then exposed to 1 nM staurosporine or vehicle for another 24 h. Next, cells were incubated with 0.5 mg/ml MTT solution (Sigma-Aldrich, Schnelldorf, Germany) for 1 h followed by MTT solution removal and addition of DMSO. Absorbance was measured at 550 nm (reference wavelength 650 nm) by using a microplate reader (FLUOstar Omega; BMG Labtech, Ortenberg, Germany). Cell viability of staurosporine-treated cells was expressed as the percentage of cell viability of vehicle-treated cells.

### Western Blotting

MDCK and NRK-52E cells were seeded into 6-well plates or T25 cell culture flasks (Greiner Bio-One, Frickenhausen, Germany) for 24 h under standard cell culture conditions. Next, cells were treated with PMA (30 nM, 24 h), non-targeting or custom-designed PKCγ siRNA (25 nM, 72 h), staurosporine (1 nM, 24 h), or vehicle only. Cells were lysed using ice-cold RIPA buffer (Cell Signaling Technology, Danvers, MA, USA) supplemented with protease and phosphatase inhibitor cocktail and EDTA (Halt, Thermo Fisher Scientific), and proteins (30 or 50 μg) were subjected to 10 or 12% SDS-PAGE and standard Western Blotting. The following antibodies were used: anti-phospho-p44/42 MAPK (Erk1/2) (Thr202/Tyr204) (#D13.14.4E), anti-p44/42 MAPK (Erk1/2) (#9102), anti-GAPDH (#D16H11) (all three from Cell Signaling Technology), clone KM2076 anti-Klotho (#SCE-KO603; Hölzel Diagnostika, Cologne, Germany), anti-PKC gamma (#PA5-28618; Invitrogen, Thermo Fisher Scientific), anti-rabbit IgG HRP-linked (Cell Signaling Technology), and anti-goat IgG HRP-linked antibody (Sigma-Aldrich). Finally, proteins were visualized using Clarity Western (Bio-Rad Laboratories) or Westar Hypernova (Cyanagen, Bologna, Italy) ECL substrate. Bands were detected by ChemiDoc MP Imaging System (Bio-Rad Laboratories) and signal intensities were determined using Image Lab Software (Version 6.1, Bio-Rad Laboratories). Data are shown as ratio of target protein to loading control glyceraldehyde-3-phosphate dehydrogenase (GAPDH) or phosphorylated protein to total protein, normalized to control.

### Statistics

Data are shown as scatter dot plots and arithmetic means ± standard deviation (SD), with *n* indicating the number of independent cell culture experiments. All data were tested for normality (Shapiro Wilk and Kolmogorov-Smirnov test). Statistical comparisons of two groups were made by two-tailed paired Student’s *t*-test or Wilcoxon matched-pairs signed rank test (for data not passing normality). For more than two groups, nonparametric Friedman test followed by Dunn’s multiple comparison test or repeated measures two-way analysis of variance (ANOVA) followed by Bonferroni’s multiple comparison test was applied as indicated in the figure legends. Only results with *p* < 0.05 were considered statistically significant. Statistical analyses were performed using GraphPad Prism 10 (version 10.0.0; GraphPad Software Inc., San Diego, CA, USA).

It was necessary to evaluate the measurements using paired tests, since they were made at different points in time and time-fixed effects are not considered. Therefore, each measurement should be treated as a group consisting of a single observation without the possibility of assuming homogeneity between groups. Consequently, there is a clear one-to-one pairing between the observations in the control and treatment groups across the entire set of observations, through the grouping that results from measuring at different times when different, uncontrollable conditions may prevail. Further, it is not possible to use unpaired testing procedures that aggregate the control and treatment group into a single representative statistic to infer on the characteristics of the sample because of non-negligible time series properties due to the design of the experiment. Aggregation may not be meaningful due to the underlying processes affecting the level of the parameters determined in the control and treatment group samples pre-treatment.

## Results

**Fig. 1:**
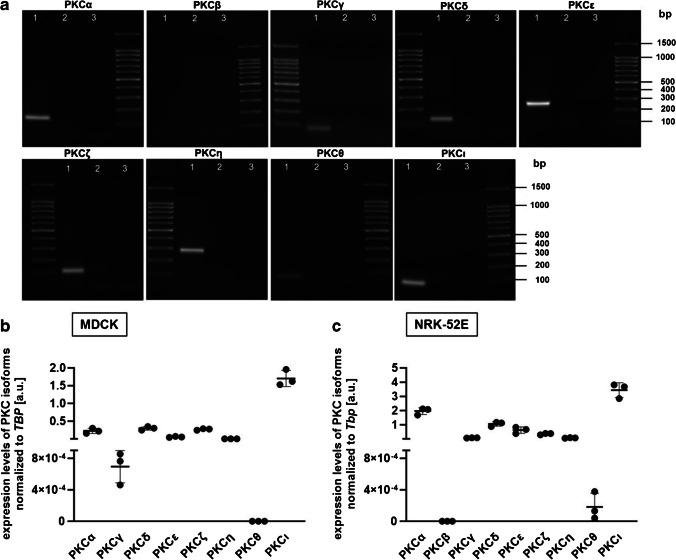
Expression of PKC isoforms in MDCK and NRK-52E cells. Original gel photographs (**a**) showing the products of PKC isoform-specific transcript amplification in untreated MDCK cells (lane 1), minus reverse transcriptase controls (lane 2), and non-template controls (lane 3). Expression levels (arithmetic means **±** SD) of PKC isoforms in MDCK (**b**, *n*=3) and NRK-52E cells (**c**, *n*=3) relative to TBP transcript levels. bp, base pairs; a.u., arbitrary units

We performed experiments in MDCK and NRK-52E cells in order to investigate whether PKC activity influences αKlotho gene expression. First, we verified the qualitative expression of different PKC isoforms in MDCK cells. According to Fig. 1a, MDCK cells expressed PKCα, PKCγ, PKCδ, PKCε, PKCζ, PKCη, PKCθ, and PKCι. After this qualitative approach, we also performed qRT-PCR analysis to compare relative expression levels of PKC isoforms in MDCK cells (Fig. 1b) and NRK-52E cells (Fig. 1c).

**Fig. 2:**
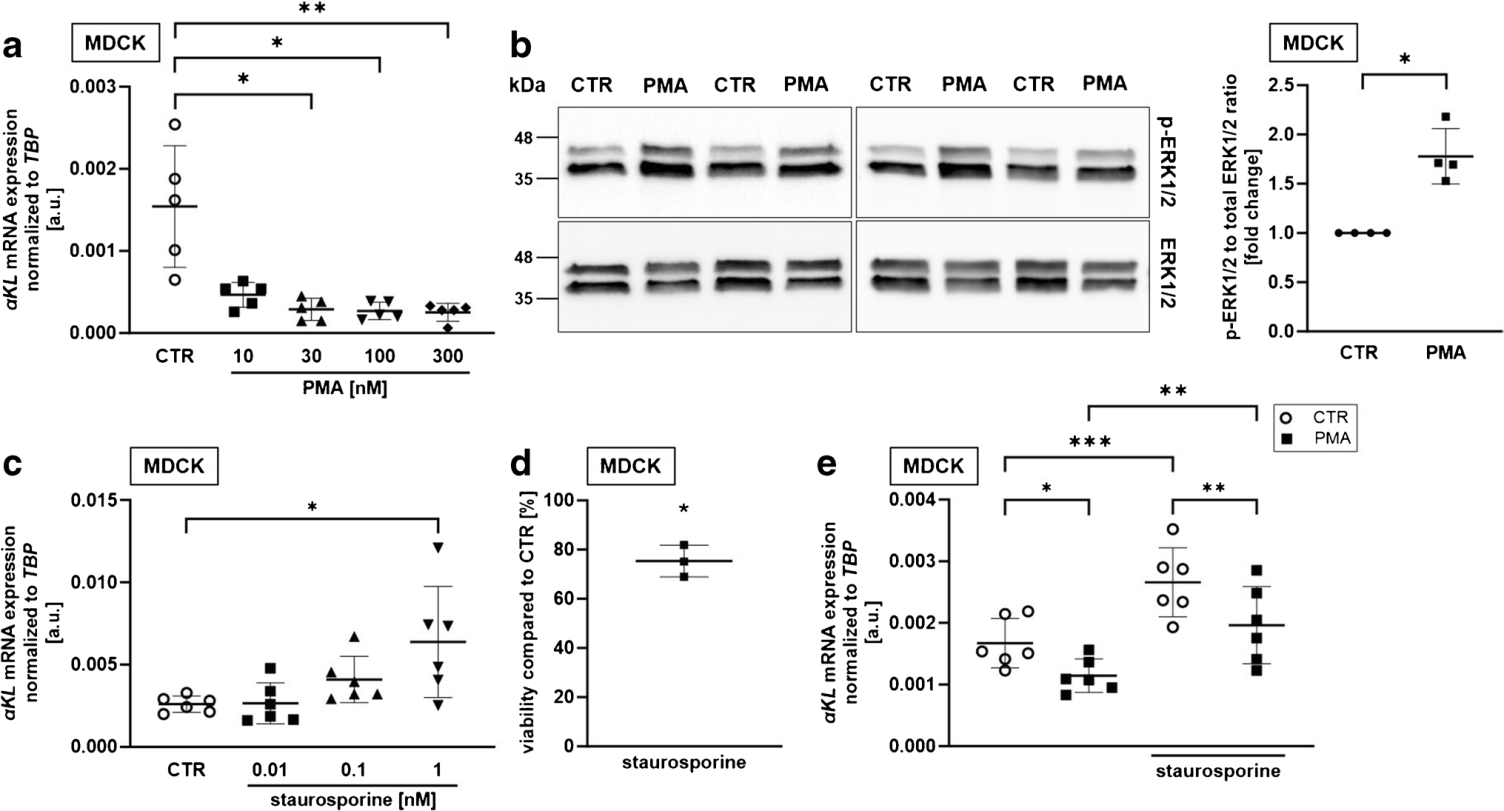
PKC activator PMA decreased and PKC inhibitor staurosporine increased αKlotho expression in MDCK cells. **a** Arithmetic means **±** SD (*n*=5) of αKlotho expression relative to TBP in MDCK cells treated with or without (CTR; white circles) PKC activator PMA. Original Western Blots showing **b** phospho-ERK(1/2) and total ERK(1/2) (left panel) and phospho-ERK(1/2) over total ERK(1/2) protein ratio (right panel; *n*=4) in MDCK cells treated with or without (CTR) 30 nM PMA for 24 h. **c** αKlotho expression relative to TBP in MDCK cells (*n*=6) treated with or without (CTR; white circles) PKC inhibitor staurosporine at the indicated concentrations for 24 h. **d** Cell viability of MDCK cells (*n*=3) treated with 1 nM staurosporine for 24 h relative to vehicle-treated cells. **e** αKlotho gene expression relative to TBP in MDCK cells (*n*=6) incubated with (black squares) or without (CTR; white circles) PKC activator PMA (100 pM) in the presence or absence of 1 nM PKC inhibitor staurosporine for 24 h. *p < 0.05, **p < 0.01, and ***p < 0.001. **a, c** Friedman test; **b, d** paired *t*-test, **e** repeated measures two-way ANOVA followed by Bonferroni’s multiple comparison test. a.u., arbitrary units

Since phorbol esters are potent stimulators of PKC, we treated the cells for 24 h with increasing concentrations of PMA and utilized qRT-PCR to quantify αKlotho transcripts [60]. As illustrated in Fig. 2a, at a dose as low as 30 nM, PMA significantly reduced transcripts specific for αKlotho in MDCK cells, an effect, even more pronounced at higher concentrations of PMA. In order to investigate whether PMA treatment indeed resulted in PKC activation, we determined phosphorylation of ERK1/2 by Western Blotting [65]. As demonstrated in Fig. 2b and suppl. Fig. 1, PMA readily upregulated phospho-ERK1/2 protein abundance, an effect in line with PKC activation. Next, we aimed to inhibit PKC activity in MDCK cells by using low concentrations of staurosporine, which may also inhibit other kinases at higher concentrations, but which has the lowest IC_50_ value for PKC of 2.7 nM [61]. Figure 2c shows that 1 nM staurosporine significantly enhanced αKlotho gene expression in MDCK cells. Treatment with 1 nM staurosporine for 24 h resulted in a moderate but significant reduction of cell viability (Fig. 2d). Another series of experiments explored whether PKC stimulation with PMA is ineffective in the presence of staurosporine. As demonstrated in Fig. 2e, PMA alone downregulated αKlotho in MDCK cells again even at a concentration as low as 100 pM in a new series of experiments, an effect, significantly blunted in the presence of staurosporine. However, even in the presence of staurosporine, PMA significantly downregulated αKlotho (Fig. 2e).

**Fig. 3:**
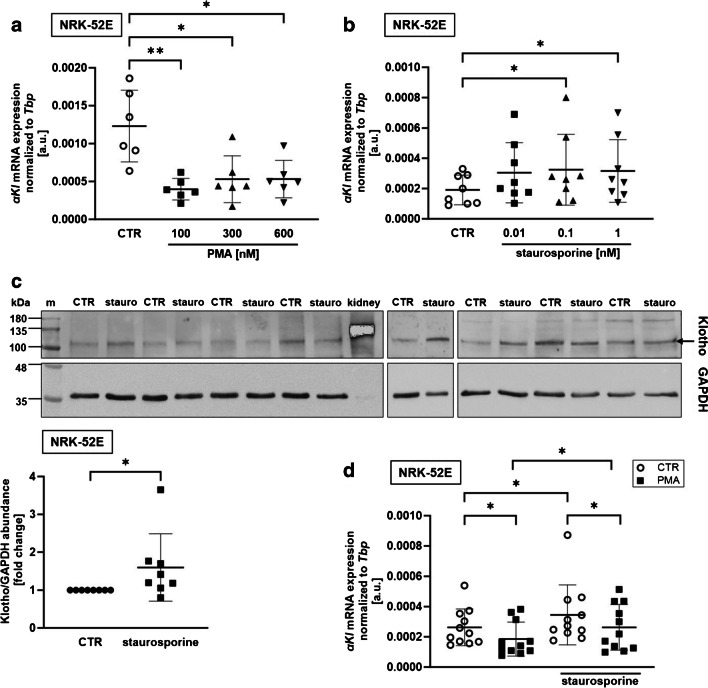
PKC activator PMA lowered and PKC inhibitor staurosporine enhanced αKlotho expression in NRK-52E cells. Arithmetic means ± SD of αKlotho expression relative to Tbp in NRK-52E cells treated with or without (CTR; white circles) **a** PKC activator PMA (*n*=6) or **b** PKC inhibitor staurosporine (*n*=8) at the indicated concentrations for 24 h. **c** Original Western Blots (upper panel) showing αKlotho and loading control GAPDH protein abundance and αKlotho over GAPDH ratio (lower panel; *n*=8) in NRK-52E cells treated with or without 1 nM staurosporine for 24 h. **d** αKlotho gene expression relative to Tbp in NRK-52E (*n*=11) cells treated with (black squares) or without (CTR; white circles) PKC activator PMA (100 nM) in the presence or absence of 1 nM PKC inhibitor staurosporine for 24 h. *p < 0.05 and **p < 0.01. **a, b** Friedman test; **c** paired *t*-test; **d** repeated measures two-way ANOVA followed by Bonferroni’s multiple comparison test. a.u., arbitrary units; m, marker; stauro, staurosporine

Next, we used NRK-52E cells to test whether PKC activity also modulates αKlotho expression in another kidney cell line. We treated these cells with PMA and found that ≥ 100 nM PMA significantly suppressed αKlotho gene expression (Fig. 3a). Similar to MDCK cells, PKC inhibition with staurosporine upregulated αKlotho mRNA abundance in NRK-52E cells, an effect reaching statistical significance at a concentration of staurosporine as low as 100 pM (Fig. 3b). Since Western Blotting did not work in canine MDCK cells, we performed Western Blotting of rat NRK-52E cell lysates in order to study whether the staurosporine effect on αKlotho gene expression also translates into higher αKlotho protein expression. As illustrated in Fig. 3c, Western Blotting of NRK-52E cell lysate using a primary anti-αKlotho antibody yielded a band in the range of 110–120 kDa that we used for quantification (Fig. 3c, lower panel). A similar band was detected using the same antibody in a mouse kidney [67] or another anti-αKlotho antibody in NRK-52E cells [76]. In a further series of experiments, PMA again downregulated and staurosporine upregulated αKlotho expression in NRK-52E cells (Fig. 3d). The combined treatment with PMA and staurosporine resulted in αKlotho expression levels not significantly different from control (Fig. 3d).

**Fig. 4:**
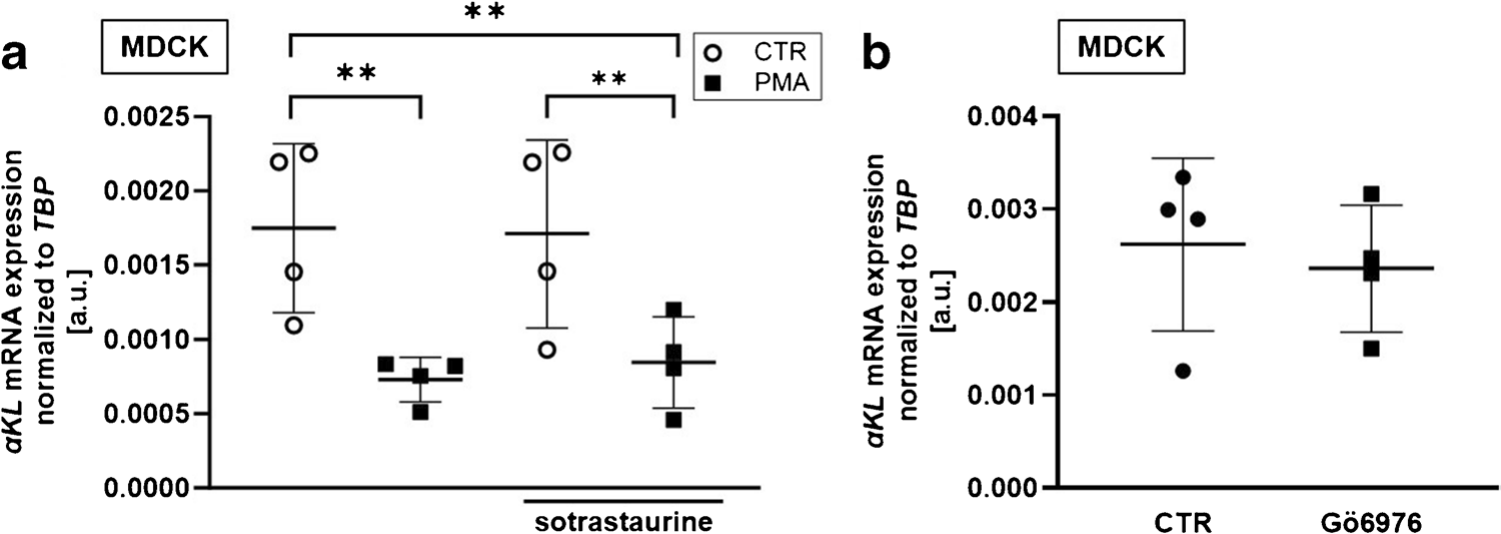
PKCα/β/δ/ε/η/θ inhibitor sotrastaurine and PKCα/β inhibitor Gö6976 did not significantly affect αKlotho expression in MDCK cells. **a** Arithmetic means ± SD (*n*=4) of αKlotho mRNA abundance relative to TBP in MDCK cells treated with (black squares) or without (CTR; white circles) PKC activator PMA (100 nM) in the presence or absence of 300 nM PKC inhibitor sotrastaurine. **b** αKlotho mRNA abundance relative to TBP (*n*=4) in MDCK cells treated with or without 40 nM PKC inhibitor Gö6976 for 24 h. **p < 0.01. **a** Repeated measures two-way ANOVA followed by Bonferroni’s multiple comparison test; **b** paired *t*-test. a.u., arbitrary units

Staurosporine is mainly an inhibitor of PKCα, PKCγ, and PKCη [41, 70]. In order to identify the PKC isoform accounting for the effect on αKlotho, we performed further experiments in MDCK cells with sotrastaurine inhibiting PKCα, PKCβ, PKCδ, PKCε, PKCη, and PKCθ, as well as with Gö6976, an inhibitor of PKCα and PKCβ. Treatment with sotrastaurine (Fig. 4a) or Gö6976 (Fig. 4b), however, failed to significantly affect αKlotho transcripts.

**Fig. 5:**
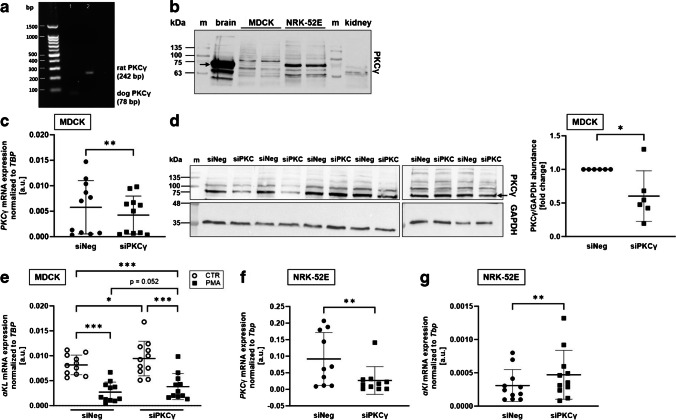
RNAi-mediated silencing of PKCγ enhanced αKlotho gene expression in MDCK and NRK-52E cells. **a** Original gel photograph showing the product of PKCγ isoform-specific transcript amplification in untreated MDCK (lane 1) and NRK-52E cells (lane 2). **b** Original Western Blots of PKCγ protein abundance in mouse brain, untreated MDCK and NRK-52E cells, and rat kidney. Arithmetic means ± SD of **c**, **f** PKCγ and **e**, **g** αKlotho gene expression relative to TBP in **c**, **e** MDCK (*n*=11) and **f, g** NRK-52E (*n*=10 and *n*=11, respectively) cells transfected with either 50 nM (NRK52E) or 25 nM (MDCK) non-targeting siRNA (siNeg) or siRNA specifically targeting PKCγ (siPKCγ) for 24 h. **d** Original Western Blots (left panel) and densitometric analysis (right panel; *n*=6) of PKCγ and loading control GAPDH in MDCK cells treated for 72 h with 25 nM non-targeting siRNA (siNeg) or siRNA targeting PKCγ. **e** αKlotho mRNA abundance relative to TBP in MDCK cells (*n*=5) treated with PKCγ-targeting or non-targeting (siNeg) siRNA in the presence (black squares) or absence (white circles) of PMA. *p < 0.05, **p < 0.01, and ***p < 0.001. **c, f, g** Wilcoxon matched-pairs signed rank test; **d** paired *t*-test; **e** repeated measures two-way ANOVA followed by Bonferroni’s multiple comparison test. a.u., arbitrary units; m, marker

Our experiments with sotrastaurine and Gö6976 prompted us to consider PKCγ as the major isoform accounting for the PKC effect on αKlotho transcripts although this isoform has thus far been thought to be mostly if not exclusively expressed in neurons [56]. As a first step, we analyzed PKCγ gene expression. As detailed in Fig. 5a, both, MDCK and NRK-52E cells, expressed mRNA specific for PKCγ, a finding verified by sequencing. Next, we performed Western Blotting to detect PKCγ protein abundance in the two cell lines. According to Fig. 5b, MDCK and NRK-52E cells exhibited a low but detectable protein expression of PKCγ. High levels of PKCγ were found in the brain and virtually no expression found in the kidney (Fig. 5b). Next, we employed RNA interference (RNAi) to silence PKCγ. According to Fig. 5c, siRNA specific for PKCγ significantly downregulated PKCγ expression in MDCK cells compared to nonsense siRNA. Also on protein level, exposure of MDCK cells to specific siRNA targeting PKCγ was paralleled by downregulation of PKCγ (Fig. 5d). Importantly, the effect was paralleled by upregulation of αKlotho gene expression (Fig. 5e). In cells treated with PKCγ-specific siRNA, the PMA effect on αKlotho was lower (*p*=0.052) than in cells treated with nonsense siRNA although PMA was still capable of suppressing αKlotho upon PKCγ silencing (Fig. 5e). Similarly, PKCγ-specific siRNA significantly downregulated PKCγ expression in NRK-52E cells (Fig. 5f) and increased the abundance of αKlotho expression (Fig. 5g). Another siRNA specifically targeting PKCγ yielded similar results in NRK-52E cells (suppl. Fig. 2).

**Fig. 6:**
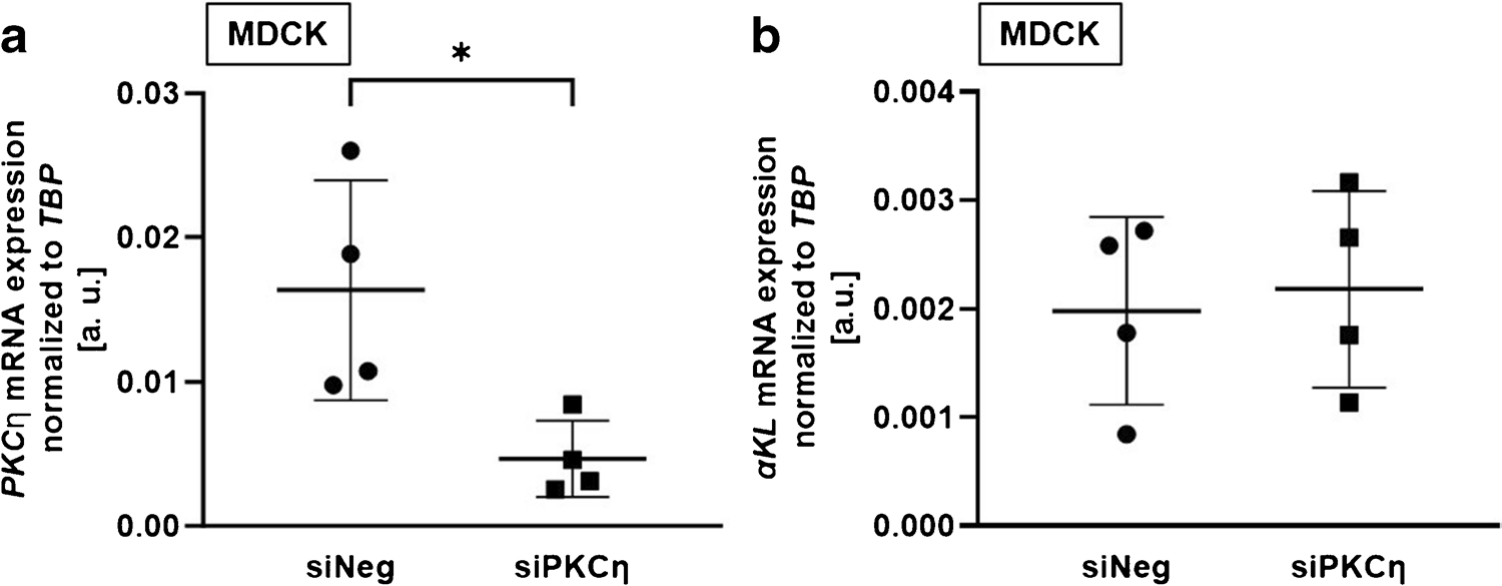
RNAi-mediated silencing of PKCη had no effect on αKlotho gene expression in MDCK cells. Arithmetic means ± SD of **a** PKCη (*n*=4) and **b** αKlotho (*n*=4) gene expression relative to TBP in MDCK cells transfected for 24 h with either non-targeting siRNA (siNeg) or siRNA specifically targeting PKCη (siPKCη). *p < 0.05. **a, b** Paired *t*-test). a.u., arbitrary units

To investigate whether isoform PKCη might also be involved, we performed further RNAi experiments. SiRNA-mediated downregulation of PKCη resulted in significantly lower PKCη expression in MDCK cells (Fig. 6a), but did not significantly affect αKlotho gene expression (Fig. 6b).

## Discussion

According to our study, PKC is a negative regulator of αKlotho gene expression in both, MDCK and NRK-52E cells. Hence, PKC activator PMA reduced αKlotho mRNA abundance in both cell lines.

Whereas our study revealed an effect of PKC signaling on αKlotho at least *in vitro*, a stimulatory effect of αKlotho on PKC activity in the kidney (and in the testis) was reported much earlier [24]. According to this study, Klotho induced PKC signaling exclusively in the kidney and testis (organs with high Klotho expression) and downregulated 25-hydroxyvitamin D_3_ 1α-hydroxylase, the key enzyme for renal 1,25(OH)_2_D_3_ production although PKC signaling directly induces 25-hydroxyvitamin D_3_ 1α-hydroxylase expression [24]. Together, these findings may suggest that Klotho and PKC signaling amplify each other in the kidney.

For our cell culture experiments, we took benefit from the varying inhibitory potentials for different PKC isoforms [31]. Staurosporine which has the lowest IC_50_ values for PKCα, PKCγ, and PKCη [41] turned out to be a potent enhancer of αKlotho expression. According to this result, it appears to be likely that the isoform most relevant for the effect is among the three. Since PKCα and PKCβ inhibitor Gö6976 and sotrastaurine inhibiting PKCα, PKCβ, PKCδ, PKCε, PKCη, and PKCθ failed to significantly affect αKlotho, we hypothesized that PKCγ is the isoform downregulating αKlotho in MDCK and NRK-52E cells. Our experiments with two different siRNAs specific for PKCγ and one for PKCη confirmed this assumption. However, we cannot rule out that isoforms other than PKCγ also impact αKlotho gene expression. This holds true in particular since PMA was still capable of significantly downregulating αKlotho in the presence of PKCγ-specific siRNA, although the PMA effect was reduced (*p*=0.052) in PKCγ-silenced cells compared to control cells. This may be due to the fact that silencing does not completely abrogate PKCγ activity or that other PKC isoforms also contribute to the PMA effect on αKlotho.

The seemingly dominant role of PKCγ for αKlotho expression in renal MDCK and NRK-52E cells comes as a surprise as PKCγ is thought to be mainly if not exclusively expressed in neurons [56]. In line with this, our Western Blotting analysis revealed a much stronger band for PKCγ in the brain than in MDCK and NRK-52E cells. However, PKCγ protein expression was detectable in MDCK and NRK-52E cells, and sequencing confirmed that both cell lines do express PKCγ mRNA. Moreover, two different siRNAs specifically targeting PKCγ yielded similar results on αKlotho expression in NRK-52E cells. Therefore, it appears to be safe to conclude that PKCγ contributes to αKlotho regulation in these renal cell lines. In the kidney, however, we detected no PKCγ expression, a finding in line with the literature according to which all PKC isoforms but PKCγ and PKCθ are expressed in the kidney [39]. A recent study, however, found strong PKCγ immunoreactivity in the proximal tubule of the developing and very weak immunoreactivity in the proximal tubule of the adult kidney [30]. Hence, it appears likely that a PKC isoform other than PKCγ is relevant for αKlotho regulation in the kidney although a role for PKCγ, at least in certain developing stages, cannot totally be ruled out. Clearly, our study suggests that care must be taken when extrapolating cell culture results to the organism.

Staurosporine is a cytotoxic agent, and treatment with staurosporine resulted in moderate reduction of viability of MDCK cells. Since reduced cell viability due to cytotoxicity has been demonstrated to ramp up αKlotho expression [47], it appears to be possible that this effect is also relevant, at least in part, for staurosporine-dependent upregulation of αKlotho. Therefore, staurosporine may also be effective independent of PKC activity. Whether these *in vitro* effects play a role *in vivo* needs to be shown. PKC-dependent gene expression is known to involve ERK, JNK/AP1, NF-κB, or JAK/STAT signaling [7]. Our study did not address the downstream signaling of the PKC effect on αKlotho. Further studies are therefore needed to elucidate whether these or other PKC-dependent pathways mediate the effect on αKlotho expression.

By Western Blotting, we could confirm in rat NRK-52E cells that PKC inhibition with staurosporine also upregulates αKlotho protein.

The regulation of αKlotho is potentially of high clinical interest since αKlotho has been revealed to exert multiple beneficial effects on a cellular level and in animal models of disease: It confers anti-inflammatory, antioxidant, antifibrotic, and tumor suppressing actions and is cardioprotective and nephroprotective or improves endothelial function [4, 14, 18, 63, 66, 71]. Not surprisingly, higher αKlotho levels are associated with better outcome in rodent models of kidney disease [40]. In human association studies, higher sKL levels are linked to better prognosis in kidney and cardiovascular disease and several malignancies [13, 15, 17, 34, 35, 50]. Therefore, enhancement of endogenous αKlotho production may be a novel and attractive therapeutic approach for those and other diseases, and therefore, a better understanding of αKlotho gene expression regulation is warranted. Our study adds to this understanding by revealing PKC as a negative regulator of αKlotho gene expression.

In line with beneficial effects of higher αKlotho availability, PKC activity is a driver of kidney disease, in particular of diabetic nephropathy, which is among the leading causes for declining kidney function ultimately resulting in end-stage renal disease [48]. Hence, PKC inhibition is beneficial in this condition [39]. Importantly, diabetic nephropathy is associated with early loss of αKlotho, and higher αKlotho levels improve the prognosis [38, 66]. Our finding, i.e., upregulation of αKlotho following PKC inhibition may contribute to the beneficial effects of this approach in diabetic nephropathy. Similarly, PKC activation has been attributed to drive pathological processes in the failing heart with different isoforms being involved [6, 57]. Also in heart disease, αKlotho is advantageous, at least in part due to its antioxidant and antifibrotic effects, and it is tempting to speculate that PKC-dependent downregulation of αKlotho as revealed by our study may, at least in part, account for unfavorable effects of PKC in heart disease [62, 72].

It must be considered, however, that therapeutic PKC inhibition may be limited by its toxicity: Staurosporine may induce neuronal death [11], and its therapeutic suitability may therefore be low. In contrast, midostaurin, another PKC inhibitor, is approved for the treatment of human disease (acute myeloid leukemia) [28]. Therefore, future studies are needed to estimate the risks and benefits of anti-PKC therapy to regulate αKlotho.

PKC-activating phorbol esters including PMA are cancerogenic due to the multiple tumor-promoting cellular effects of PKC [21, 45]. In our study, PMA lowered expression of potent tumor suppressor αKlotho, an effect which may also be relevant for the cancer-promoting effects of PKC activators.

It has to be kept in mind that our study is solely based on cell culture experiments involving two different kidney cell lines. Future studies are needed to decipher the significance of PKC for αKlotho regulation *in vivo*.

In summary, our study uncovers PKC activity as a potent negative regulator of αKlotho gene expression in MDCK and NRK-52E cells with PKCγ having a pivotal role for this effect in the two cell lines. PKC-dependent suppression of αKlotho may be relevant for heart and kidney disease as well as cancer progression.

### Supplementary information

Below is the link to the electronic supplementary material.

ESM 1 (DOCX 802 KB)

## Data Availability

The datasets generated during and/or analyzed during the current study are available from the corresponding author on reasonable request.
