# Supplementary material for: PKC regulates αKlotho gene expression in MDCK and NRK-52E cells
Source: Pflugers Arch. 2023 Sep 29;476(1):75–86. doi: 10.1007/s00424-023-02863-3 (PMC10758369; doi:10.1007/s00424-023-02863-3)
Supplement: Supplementary file 1 — ESM 1 (DOCX 802 KB) [file 424_2023_2863_MOESM1_ESM.docx]

**Supplementary methods**

*Silencing*

Silencing of NRK-52E cells was carried out as detailed in the main text. Incubation time was prolonged to 48 h. The following custom-designed PKCγ siRNA (50 nM; Invitrogen) was used:

sense: AAAUGUUCCUGUUGCGAAAtt.

**Supplementary Figures**


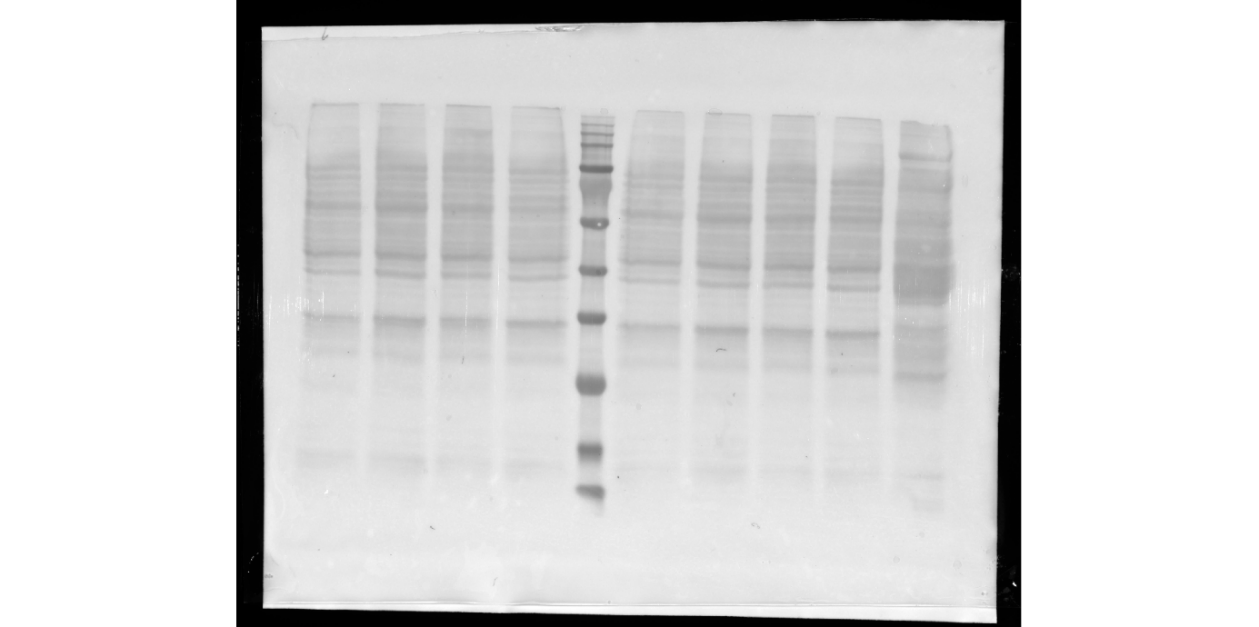

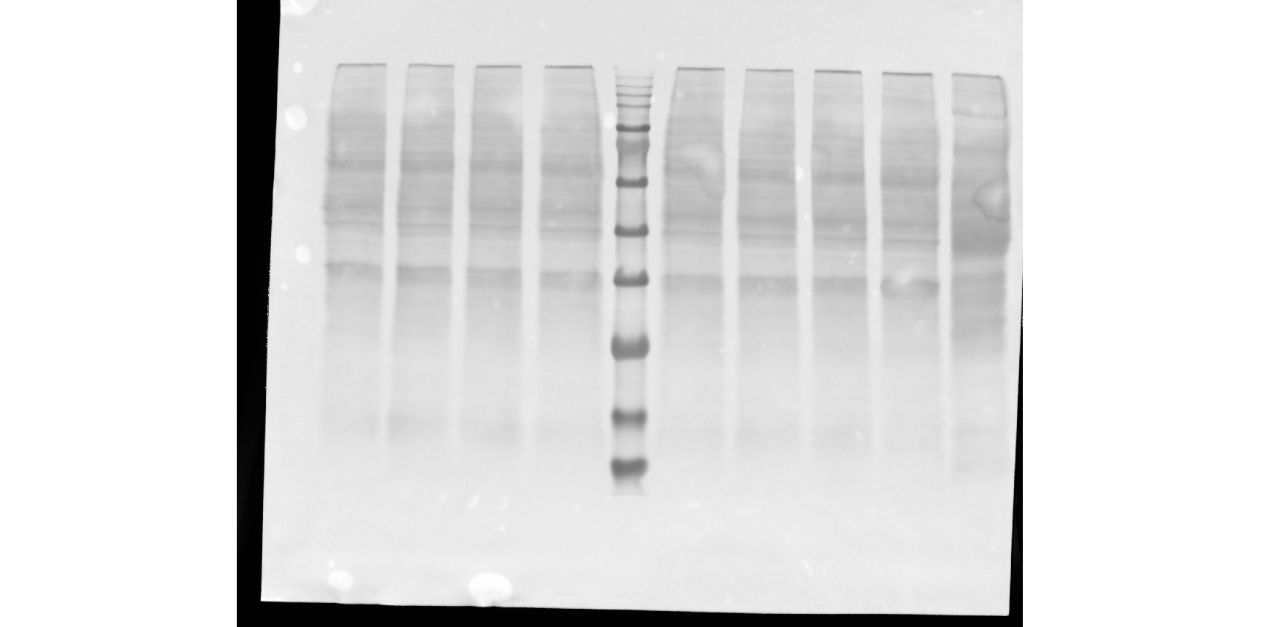


**total ERK(1/2)**

**total ERK(1/2)**

**phospho-ERK(1/2)**

**phospho-ERK(1/2)**

**Suppl. Fig. 1: Total protein staining of phospho-ERK(1/2) and total ERK(1/2) Western Blots**

Original photographs showing total protein staining (Ponceau S) of phospho-ERK(1/2) and total ERK(1/2) Western Blots.

**b**

**a**

**NRK-52E**

**NRK-52E**

**Suppl. Fig. 2: RNAi-mediated silencing of PKCγ enhanced αKlotho gene expression in NRK-52E cells**

Arithmetic means ± SD of **a** PKCγ (n=6) or **b** αKlotho (n=6) gene expression relative to Tbp in NRK-52E cells transfected for 48 h with either non-targeting siRNA (siNeg) or siRNA specifically targeting PKCγ (siPKCγ). *p < 0.05. **a** Paired *t*-test, **b** Wilcoxon matched-pairs signed rank test. a.u., arbitrary units.
